# Effects of Crocetin Esters and Crocetin from *Crocus sativus* L. on Aortic Contractility in Rat Genetic Hypertension

**DOI:** 10.3390/molecules200917570

**Published:** 2015-09-22

**Authors:** Silvia Llorens, Andrea Mancini, Jessica Serrano-Díaz, Anna Maria D’Alessandro, Eduardo Nava, Gonzalo Luis Alonso, Manuel Carmona

**Affiliations:** 1 Department of Medical Sciences, School of Medicine and Regional Centre for Biomedical Research (CRIB), University of Castilla-La Mancha, Albacete 02006, Spain; E-Mails: Silvia.Llorens@uclm.es (S.L.); Eduardo.Nava@uclm.es (E.N.); 2 Department of Life, Health and Environmental Sciences, University of L’Aquila, L’Aquila 67100, Italy; E-Mails: mancio_1982@hotmail.com (A.M.); anna.dalessandro@cc.univaq.it (A.M.D.); 3 School of Agricultural Engineering, University of Castilla-La Mancha, Albacete 02071, Spain; E-Mails: Jessica.Serrano@uclm.es (J.S.-D.); Gonzalo.Alonso@uclm.es (G.L.A.); 4 Albacete Science and Technology Park, Paseo de la Innovación 1, Albacete 02006, Spain

**Keywords:** crocetin, crocetin esters, crocins, endothelium, hypertension

## Abstract

Background: Endothelial dysfunction, characterized by an enhancement in vasoconstriction, is clearly associated with hypertension. Saffron (*Crocus sativus* L.) bioactive compounds have been recognized to have hypotensive properties. Recently, we have reported that crocetin exhibits potent vasodilator effects on isolated aortic rings from hypertensive rats. In this work, we have aimed to analyze the anticontractile ability of crocetin or crocetin esters pool (crocins) isolated from saffron. Thus, we have studied the effects of saffron carotenoids on endothelium-dependent and -independent regulation of smooth muscle contractility in genetic hypertension. Methods: We have measured the isometric responses of aortic segments with or without endothelium obtained from spontaneously hypertensive rats. The effects of carotenoids were studied by assessing the endothelial modulation of phenylephrine-induced contractions (10^−9^–10^−5^ M) in the presence or absence of crocetin or crocins. The role of nitric oxide and prostanoids was analyzed by performing the experiments with L-NAME (NG-nitro-l-arginine methyl ester) or indomethacin (both 10^−5^ M), respectively. Results: Crocetin, and to a minor extent crocins, diminished the maximum contractility of phenylephrine in intact rings, while crocins, but not crocetin, increased this contractility in de-endothelizated vessels. In the intact vessels, the effect of crocetin on contractility was unaffected by indomethacin but was abolished by L-NAME. However, crocetin but not crocins, lowered the already increased contractility caused by L-NAME. Conclusions: Saffron compounds, but especially crocetin have endothelium-dependent prorelaxing actions. Crocins have procontractile actions that take place via smooth muscle cell mechanisms. These results suggest that crocetin and crocins activate different mechanisms involved in the vasoconstriction pathway in hypertension.

## 1. Introduction

Crocetin esters and crocetin (CCT), carotenoids present in the dried stigmas of *Crocus sativus* L. (saffron), are considered the pharmacologically active components of saffron [1]. These have been shown to possess protective effects against cardiovascular diseases [2,3,4,5], neurological disorders [6,7], cancer [8,9,10], and hemorrhagic shock [11]. Regarding the cardiovascular effects of saffron, previous studies revealed that this spice or its constituents possess hypotensive effects [12,13,14,15,16]. These studies showed that extracts of saffron petals reduced mean arterial blood pressure in rats. This effect appeared to be related to the inhibitory effect of the extract on vascular smooth muscle, via blockade of calcium channels, inhibition of sarcoplasmic reticulum Ca^2+^ release into the cytosol [13,17] or via antagonism of adrenergic receptors [14]. Also, hypotensive effects have been reported for aqueous and ethanol extracts of saffron stigmas and for two active constituents of this plant, crocin and safranal [15,16]. On the other hand, CCT has been reported to possess antioxidant activity [18] and to increase the activity of endothelial nitric oxide synthase [19].

Hypertension develops when peripheral vascular resistance increases and this can be caused by an abnormal vasoconstriction of the vasculature [20,21]. In physiological conditions vessels are exposed to a basal vasoconstriction or vascular tone which is counterbalanced by substances released by vascular endothelial cells [20,21]. Thus, the endothelium regulates vascular tone by the synthesis and release of vasodilators such as nitric oxide (NO), prostacyclin (PGI2), and endothelium-derived hyperpolarizing factor (EDHF). However, endothelial cells also produce vasoconstrictors such as endothelins, prostanoids (such as thromboxane A2), and oxygen reactive species. An imbalance between endothelial vasoconstrictors and vasodilators is considered the hallmark of endothelial dysfunction and is clearly related to hypertension [22].

Although hypotensive effects and inhibition of vascular smooth muscle contractility have been reported for saffron or its constituents, there is no data on the possible effects on the endothelial modulation of vascular contraction in hypertension. In the present work we hypothesized that the beneficial effects of aqueous extracts of saffron stigmas in hypertension could be partly related to an improvement in the ability of the endothelium to counteract vascular smooth muscle contraction. We have tested saffron biocompounds which have reported vasorelaxant effects: pools of crocetin esters and CCT [23]. We have measured the acute effects of these compounds on the vasoconstriction elicited by phenylephrine in aortic segments from genetically hypertensive rats (SHR). This was performed, in the presence or absence of indomethacin (an inhibitor of cyclooxygenase) or L-NAME (an inhibitor of NO synthase) to evaluate the possible interactions of crocetin esters and CCT with endothelial prostanoids or NO.

## 2. Results

### 2.1. Analysis of Crocins

Organoleptic properties (color, taste, and aroma) of saffron are due to crocetin esters, picrocrocin and safranal, respectively. It is important to characterize the saffron used in studies in order to determine its quality. This is correlated with its amount in crocetin esters, picrocrocin, and safranal, because these compounds are also responsible for its bioactive properties. The chromatographic purity of the compounds identified as crocetin esters, safranal, and picrocrocin correspond to the 99.9% absorbance at 440 nm, 95.3% at 330 nm, and 96.1% at 250 nm, respectively. Crocins were isolated from saffron to yield 25.5%.

**Figure 1:**
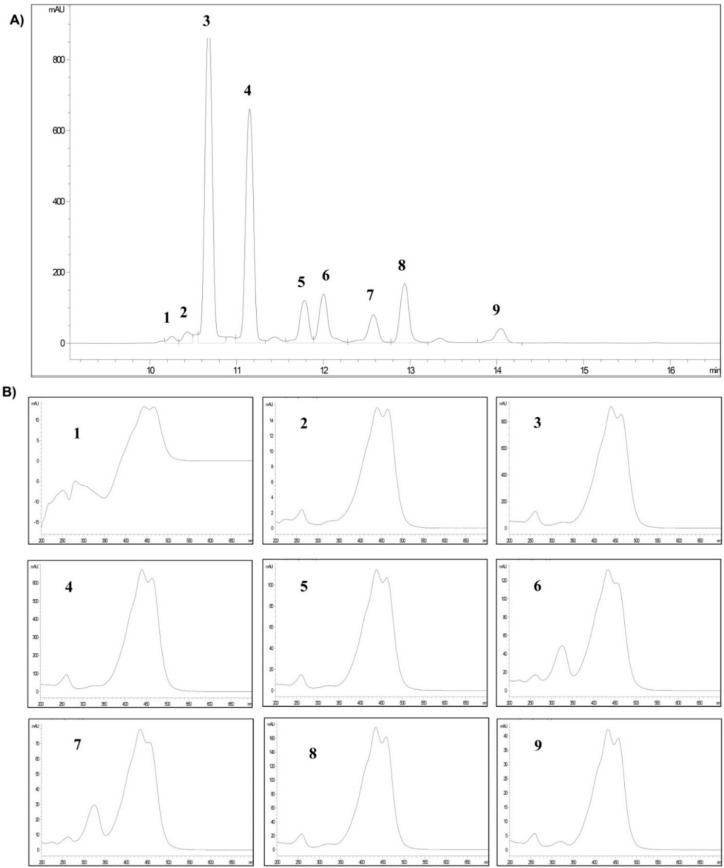
(**A**) Chromatogram at 440 nm of the crocetin esters pool (crocins); (**B**) UV-vis spectrum each.

The crocetin esters identified in saffron spice are shown in Figure 1. Their retention times (*t*_R_) at 440 nm and UV-vis spectra of each one (Figure 1A,B, respectively), were consistent with those reported by Carmona *et al.* [24], (Table 1).

**Table 1:** Peak number, retention time (*t*_R_) and area percentage each chromatographic peak corresponding to crocetin esters (crocins) identified in the pool of crocetin esters.

Crocetin Esters (*)	Peak	(*t*_R_) (min)	Area (%)
*trans*-5tG	1	10.252	0.9
*trans*-5nG	2	10.429	1.6
*trans*-4GG	3	10.675	38.6
*trans*-3Gg	4	11.148	29.4
*trans*-2gg	5	11.781	6.2
*cis-*4GG	6	12.001	7.3
*cis-*4ng	7	12.575	4.8
*trans*-2G	8	12.936	8.6
*cis-*3Gg	9	14.047	2.6

(*) Meaning of each letter in the abbreviation of the name of each crocetin esters: Number (5, 4, 3, 2)— number of glucose molecules attached to the molecule of crocetin; t—triglucose ; G—gentiobiose; g—glucose; n—neapolitanose.

The main crocetin ester was *trans*-4GG (38.6% of the total content), followed by *trans*-3Gg, *trans*-2G, *cis*-4GG, *trans*-2-gg and *cis*-3Gg. *Cis* isomers showed an additional absorption band around 324 nm in their UV-vis spectra (Figure 1B, spectra: 6, 7, 9).

Regarding the proportion of *cis*/*trans*-crocetin esters, the chromatographic area of the *trans*-crocetin esters accounted for 85.3% of the total chromatographic area at 440 nm.

### 2.2. Myographical Results

**Figure 2:**
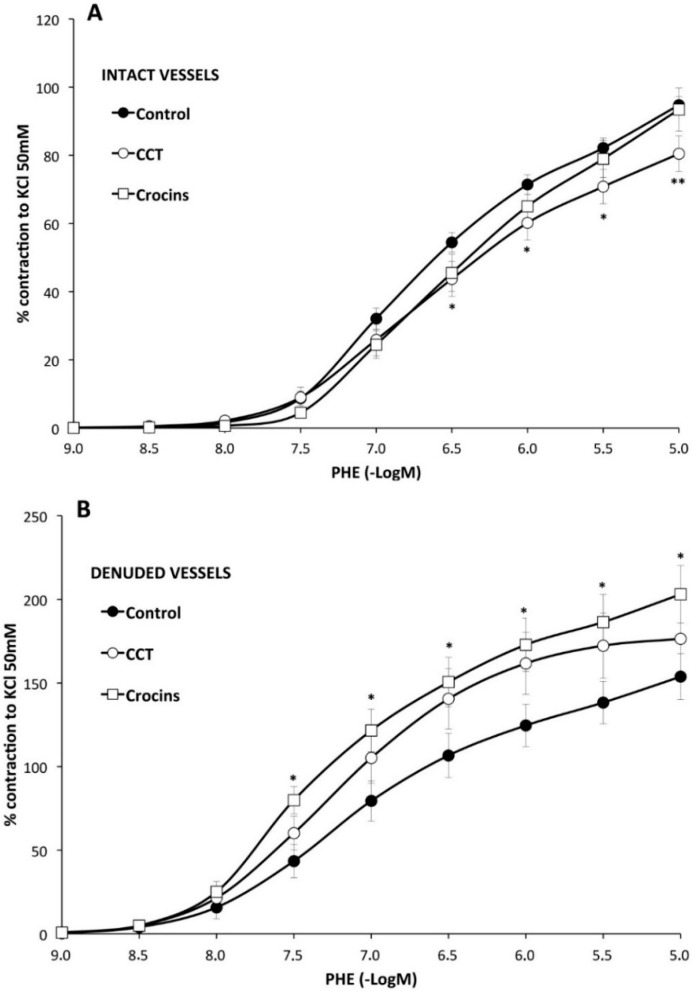
The effect of crocetin (CCT) and crocins on the endothelial modulation of aortic smooth muscle contraction elicited by phenylephrine was assessed by performing a dose-response curve in the presence (panel **A**) or absence (panel **B**) of endothelium (please note the different scale in both panels). The existence of an intact endothelium is required for CCT, but not for crocins, to diminish phenylephrine responses. * *p* < 0.05, ** *p* < 0.01 compared with the control curve.

Effects of saffron carotenoids on the endothelial modulation of vascular smooth muscle contraction. The presence of CCT in the organ bath diminished by 15% PHE-mediated contractility of the vessels (Figure 2A), as seen by the lower *E*_max_ value (80 ± 5% of KCl contraction *vs.* 95 ± 3%) (Table 2).

Crocins, instead, did not modify the *E*_max_ (93 ± 6%) elicited by PHE but diminished the sensitivity to the catecholamine (pD_2_ value: 6.0 ± 0.1 *vs.* 6.28 ± 0.04) (Table 2). These results indicate that CCT diminishes the contractility of the vessels to PHE, while crocins exhibit minor anticontractile effects.

As expected, de-endothelialized segments, compared with intact segments, elicited a stronger contraction (154 ± 14%) and showed increased sensitivity (pD_2_, 6.6 ± 0.1) to PHE (Table 2) indicating that the endothelium of the SHR aorta is functional and able to counteract PHE-mediated vasoconstriction.

**Table 2:** Acute effects of saffron carotenoids on vascular responses.

	Control			with CCT			with Crocins		
	*E*_max_ (%)	pD_2_	*n*	*E*_max_ (%)	pD_2_	*n*	*E*_max_ (%)	pD_2_	*n*
PHE	95 ± 3	6.28 ± 0.04	26	80 ± 5 **	6.2 ± 0.1	10	93 ± 6	6.0 ± 0.1 *	10
PHE rubbed	154 ± 14 ^a^	6.6 ± 0.1 ^a^	8	176 ± 23 ^bb^	6.78 ± 0.06 ^bb^	8	203 ± 17 *^,cc^	6.82 ± 0.02 ^cc^	7
PHE + Indo	66 ± 3 ^aa^	6.22 ± 0.06	10	74 ± 6	6.2 ± 0.1	8	65 ± 7 ^cc^	6.13 ± 0.06	8
PHE + L-NAME	128 ± 5 ^aa^	6.5 ± 0.1 ^aa^	6	112 ± 2 *^,bb^	6.3 ± 0.1	6	121 ± 3 ^cc^	6.4 ± 0.1 ^cc^	6

* *p* < 0.05, ** *p* < 0.01 compared with the corresponding curve in the control column. ^a^ *p* < 0.05, ^aa^ *p* < 0.01 compared with phenylephrine alone. ^bb^ *p* < 0.01 compared with phenylephrine with CCT. ^cc^ *p* < 0.01 compared with phenylephrine with crocins. “*n*” stands for the number of segments. Not every possible comparison has been analyzed.

In these de-endothelialized vessels, CCT did not significantly modify the *E*_max_ nor the pD_2_ values obtained with PHE alone (Figure 2B, Table 2). The addition of crocins, in contrast, caused a significantly higher maximal contraction (203 ± 17%) compared to that obtained with PHE alone (Figure 2B, Table 2).

These results suggest that CCT possesses anticontractile ability when endothelium is present and crocin esters procontractile effects when the endothelium is removed. De-endothelialized vessels in the presence of CCT exhibit a trend, which did not reach significance, to contract with a higher strength.

**Figure 3:**
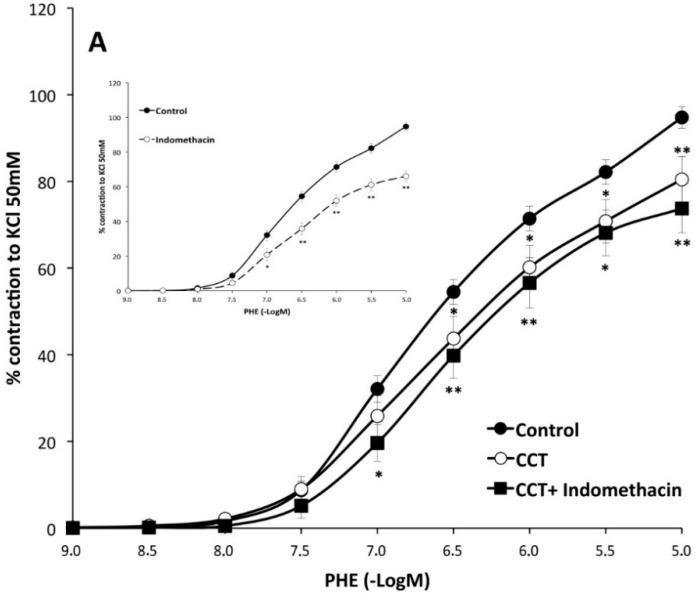

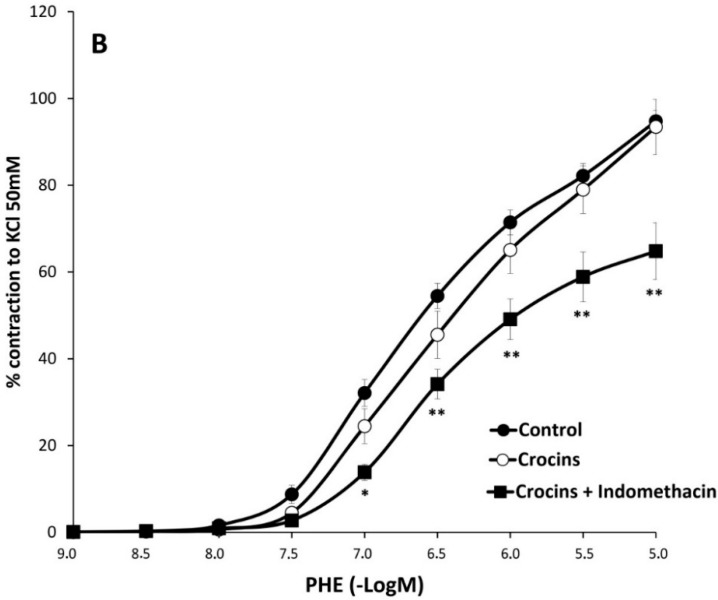
Effects of crocetin (CCT) (panel **A**) and crocins (panel **B**) on the responses of aortic segments to increasing concentrations of phenylephrine in the presence or absence of indomethacin. Inset figure shows the effects of indomethacin alone on phenylephrine contractions. Inhibition of the cyclooxygenase pathway reduces contractions elicited by phenylephrine. * *p* < 0.05, ** *p* < 0.01 compared with the control curve.

The role of endothelial prostaglandins on the action of saffron carotenoids. Treatment with indomethacin lowered the maximum effect of PHE (66 ± 3%) (Figure 3 inset). Incubation, of crocins or CCT-treated vessels, with indomethacin also diminished the ability of PHE to contract but did not modify the responses obtained with indomethacin alone (Table 2). Addition of indomethacin to CCT-treated vessels did not further modify the *E*_max_ (74 ± 6%) obtained by CCT alone on PHE-vasoconstriction. In contrast, addition of indomethacin to the vessels incubated with crocins elicited a marked reduction of the *E*_max_ obtained by crocins alone on PHE contractility (65 ± 7%) (Figure 3A,B and Table 2). These results suggest that CCT possesses the ability to lower smooth muscle cell contractility like indomethacin and this is dependent on the presence of endothelium. In contrast, crocins does not lower contractility unless COX is inhibited, suggesting that this effect is produced by indomethacin.

**Figure 4:**
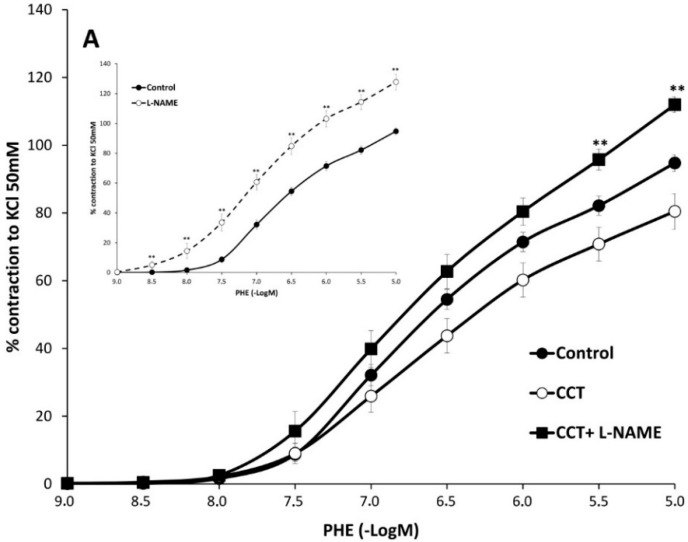

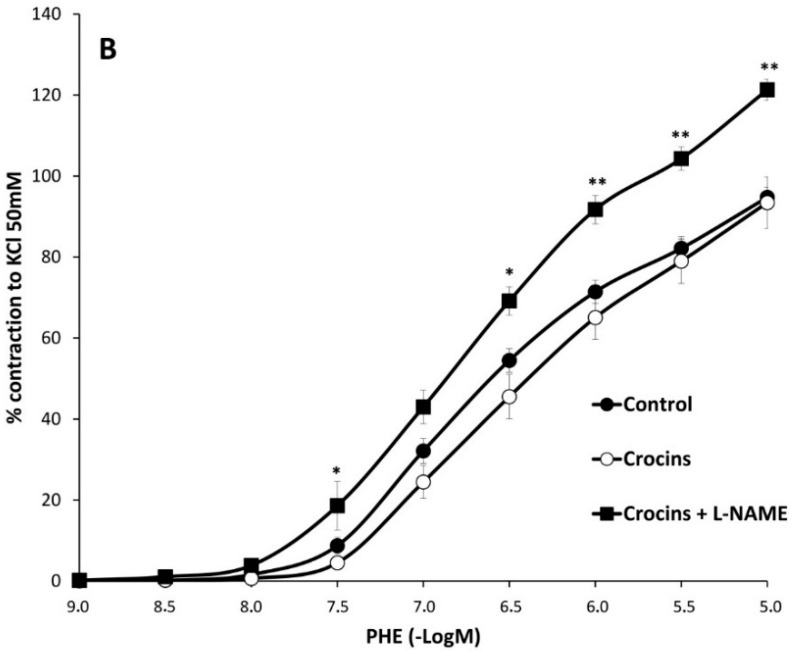
Effects of L-NAME on the responses of crocetin (CCT) (panel **A**) and crocins (panel **B**) -preincubated aortic segments to increasing concentrations of phenylephrine. Inset figure shows the effects of L-NAME alone on phenylephrine contractions. When NO synthesis was inhibited, responses to phenylephrine were increased in vessels treated with carotenoids. * *p* < 0.05, ** *p* <0.01 compared with the CCT or crocins curve.

Role of NO on the action of saffron carotenoids. NO synthase inhibition with L-NAME (10^−5^ M) enhanced both the *E*_max_ (128 ± 5%) and pD_2_ (6.5 ± 0.1) value of PHE contraction of the vessels (Figure 4 inset). The presence of L-NAME in crocins or CCT-treated segments similarly, increased PHE-induced contractions and reverted the anticontractile effect of CCT (Figure 4A,B and Table 2) suggesting that CCT acts partially via NO. The increased contractions of L-NAME-treated segments were slightly, but significantly, by addition of CCT (but not by addition of crocins (Table 2). This suggests that CCT elicits vasorelaxant effects independent of NO.

## 3. Discussion

We have examined the effects of crocetin and crocins isolated from saffron stigma on endothelial function of the aorta of genetically hypertensive rats to test the hypothesis that the cardiovascular beneficial effects of these biocompounds are related to actions on the endothelial control of the vascular hyperreactivity of hypertension. In an attempt to clarify how CCT and crocins alter this function, we carried out experiments aimed to analyze whether these biocompounds affect the endothelial modulator responses to the vasoconstriction produced with phenylephrine. We tested this catecholamine because overreactive vessels in the context of an elevated sympathetic tone is a hallmark of hypertension [25]. Our experiments showed that CCT, but not crocins diminish the maximal vessel response to the vasoconstrictor. Although this inhibition of PHE contraction by CCT (15%) could be considered small, it is important that this compound has the ability to diminish vasoconstriction in hypertension, because this pathology is fundamentally caused by an enhanced vasoconstriction. In this context, the magnitude of this inhibition is very remarkable. Crocins marginally affected the sensitivity of the vessel to PHE as measured by the pD_2_ indicating a minor anticontractile effect at low concentrations. The effect of CCT can be explained by several properties of this compound. For example, it has been reported that CCT possesses antioxidant activity properties caused [7,26,27,28] by activation of endogenous antioxidant enzymes such as superoxide dismutase and glutathione peroxidase [7,29]. Also, CCT has a direct ROS scavenging effect [30]. Additionally, CCT can increase endothelial nitric oxide synthase activity [19]. We believe that the decreased adrenergic response observed in the presence of CCT was mediated by actions of this bioactive compound on endothelial cells because when the vessel was de-endothelialized, CCT did not show the same effects. This suggests that substances released by the endothelial layer are responsible for the anticontractile response obtained of CCT. This result complements our findings recently published [23] which showed that the aqueous CCT extract improves the relaxation induced by acetylcholine in aorta from hypertensive rats through endothelium and NO-dependence.

We have attempted to elucidate which endothelial substances might be affected by CCT and crocins. Indomethacin experiments were aimed to study a possible role of endothelial prostanoids. These experiments showed that the incubation of the vessels with indomethacin caused a reduction of the contraction. This was expected since it is well known that the hyperreactivity of hypertensive vessels is partly caused by vasoconstrictive prostaglandins [31]. The addition of indomethacin to CCT-treated arteries did not modify the beneficial action of this saffron compound, which primarily would discard a dependency on the cyclooxygenase pathway. It is of note that the effects of CCT and those of CCT in the presence of indomethacin were undistinguishable. If these two compounds acted by different mechanisms one would expect an addition of effects and consequently, an increase of the response of CCT plus indomethacin with respect to CCT alone. This suggests that CCT, at least partially, does have an inhibitory effect on COX. On the other hand, the anticontractile effect of crocins was so marginal that the effect observed in the presence of indomethacin was likely due solely to prostaglandin inhibition. We hypothesized that CCT may be stimulating other vasodilator substances from the endothelium. NO is the major endothelial vasodilator, especially in large arteries such as the aorta [32]. To test whether NO is involved in the action of CCT, we carried out experiments using L-NAME. The anticontractile effects of CCT were completely abolished when NO synthase was inhibited with L-NAME, suggesting that the action of these compounds is partly caused by NO. L-NAME alone elicited an increase in the contractile responses of the aortic segments comparable to that seen in rubbed segments, thus confirming the existence of a basal release of NO. Interestingly, addition of L-NAME to CCT, but not when the vessels were treated with crocins, diminished the contraction produced by L-NAME alone on PHE responses. This suggests that the action of CCT on endothelial physiology is able to compensate the lack of NO possibly by stimulating the release of other endothelial vasodilators. As stated before, when endothelium was removed, the presence of crocins augmented the force generated by PHE on aortic segments. This was not found with CCT. Thus, it appears that specifically crocins, but not CCT, have a contractile action on the vessel wall. Other researchers, such as Imenshahidi *et al.* [15] have shown a hypotensive effect of acute administration of saffron or various of its compounds, including crocin (refers to a crocetin ester) [15]. Experiments performed in isolated tissues reveal that crocin inhibits calcium currents and relaxes smooth muscle cells of trachea [12], vas deferens, and ileum [14]. This contrasts with our present results and the discrepancy might be due to tissue differences in the effects of crocins. Another possibility might be due to the involvement in vascular tissue of potassium channels. While inhibition of calcium currents leads to relaxation, inhibition of potassium currents depolarizes and increases contractility.

Overall, our results show that CCT and crocins differentially affect vascular contractility in hypertension. CCT has prorelaxing actions through endothelial cells, while the main target of crocins are smooth muscle cells, leading to contractile effects when endothelium is absent. Thus, the vascular actions of saffron compounds are heterogeneous and the final effect on blood pressure is probably the result of the synergic action of all the chemical constituents of the plant.

In summary, we have shown: (a) CCT, and to a lesser extent crocins, diminish PHE contractility in an endothelium-dependent manner; (b) CCT, when the endothelium is absent, stimulates this contractility; (c) the endothelial actions of CCT are partially dependent on NO; (d) CCT, but not crocins, reveal vasorelaxing properties in full NO blockade conditions. Finally, (e) CCT may have an inhibitory effect on COX, in this sense further investigations need to be done by combining different concentrations of CCT and indomethacin.

## 4. Experimental Section

### 4.1. Plant Material

We used dry stigmas of pure “Azafrán de La Mancha” (La Mancha saffron), a protected designation of origin (PDO) which has a guaranteed coloring strength >200. When this parameter, which represents the color strength, exceeds 200 units indicates that saffron belongs to the best commercial saffron quality (Category I) [33]. Saffron was directly purchased from a reputed producer (Agrícola Técnica de Manipulación y Comercialización in Minaya, Albacete, Spain) and stored in dark conditions at 4 °C until further use.

### 4.2. Isolation CCT and Crocetin Esters

CCT was isolated and analyzed as described in Mancini *et al.* [23].

To isolate the pool of crocetin esters (from this point on refer to “crocins”), 5.0014 g of saffron were put into an Erlenmeyer flask with 400 mL of hexane with stirring for 16 h, thereafter filtered and the residue vacuum dried. Subsequently, this residue was extracted with 400 mL of water at room temperature with stirring for 1 h. The supernatant was chromatographed on a preparative low-pressure column (VersaFlash, Supelco, Bellefonte, PA, USA). C18 Cartridges (80 mm × 150 mm) were used. First, 400 mL of an acetonitrile solution (ACN) at 5% were added, then ACN at 15% to totally elute picrocrocin, finally crocins were subsequently eluted with pure ACN. The solvent was removed under vacuum at 35 °C, obtaining 1.2755 g of crocins which were stored in the dark at −17 °C until further use.

### 4.3. High-Performance Liquid Chromatography with Photodiode Array Detection (HPLC-DAD) Analysis

Twenty microliters of crocin solution (10 mg of crocins in 100 mL of water) was injected into an Agilent 1200 HPLC (Palo Alto, CA, USA) equipped with a 150 mm × 4.6 mm (internal diameter), 5 μm Phenomenex (Le Pecq Cedex, France) Luna C18 column that was equilibrated at 30 °C. The eluents were water (A) and ACN (B) with the following gradient: 20% B, 0–5 min; 20%–80% B, 5–15 min; and 80% B, 15–20 min. The flow rate was 0.8 mL/min. The DAD detector (Hewlett Packard, Waldbronn, Germany) was set at 250, 330, and 440 nm for picrocrocin, safranal and crocetin esters detection, respectively. All analyses were performed in duplicate, and two measurements were performed for each replicate. Crocins (*trans*-5tG, *trans*-5nG, *trans*-4GG, *trans*-3Gg, *trans*-2gg, *cis*-4GG, *cis*-4ng, *trans*-2G and *cis*-3Gg crocetin ester) were identified according to Carmona *et al.* [24], and quantified according to Sánchez *et al.* [34].

### 4.4. Myographical Methods

For a model of hypertension, 12–14-week old male spontaneously hypertensive rats (SHR) were used (*n* = 14). These were obtained from Charles River Laboratories (Barcelona, Spain), housed in an open system room with a one-way airflow system (temperature, 20–22 °C; light period, 12/12; humidity, 45%), and were given a commercial diet and tap water *ad libitum*. All procedures were carried out in accordance with the Declaration of Helsinki and Spanish Real Decreto 1201/2005 on Protection of Animals Utilized for Experimentation and Other Scientific Purposes.

Isometric tension was determined as described in Mancini *et al.* [23] and Llorens *et al.* [35]. After euthanizing the rats by CO_2_ inhalation, the aorta was carefully dissected with microscissors under a microscope. The isolated thoracic aorta was then placed in ice-cold Krebs-Henseleit solution (KHS). Blood and adherent fat were cautiously cleaned and the artery cut in sequential 3.3 mm segments. These segments were immersed in a 5 mL organ bath containing 37 °C KHS bubbled with 95% O_2_ and 5% CO_2_ to provide a pH of 7.3–7.4. The composition of KHS was (in mM): NaCl, 115; KCl, 4.6; CaCl_2_, 2.5; KH_2_PO_4_, 1.2; MgSO_4_, 1.2; NaHCO_3_, 25; EDTA, 0.01; and glucose, 11.1. Two stainless steel L-shaped pins (200 μm in diameter) were introduced through the arterial lumen under a microsurgery microscope. Isometric tension was determined with two 4-channel myographs (Danish MyoTechnology, Aarhus, Denmark) and recorded on a PowerLab unit (ADInstruments, Castle Hill, Australia). A resting tension of 2.5 g was applied to the aortic segments and they were held for an equilibration period of 60 min before the experiments were started. Tension was readjusted and the bath fluid changed when necessary. Next, the vascular reactivity was checked by depolarization with a high K^+^ solution (same composition as KHS but containing 50 mmol/L KCl). Tissues producing less than 1.5 g in force were discarded. The endothelial layer integrity was assessed with a single dose of acetylcholine (ACH, 10^−6^ M) in each ring after a stable contraction was obtained with phenylephrine (PHE, 10^−5^ M). Intact vessels failing to achieve at least 60% relaxation with this dose of acetylcholine were assumed to be damaged and were discarded. To remove the endothelium, proper rubbing of the intimal layer was tested by the lack of relaxation on application of the mentioned dose of ACH. Segments passing these tests were used for the pharmacological experiments. To better compare results between segments, all results were normalized to a percentage of the force achieved by PHE (10^−5^ M) in each particular aortic segment, except for the dose-response curves to phenylephrine which were normalized to the KCl contraction. In every protocol, crocins and CCT were added to the bath 30 min prior to protocols so as to reach a concentration of 1.2 × 10^−5^ M. Subsequently, PHE was added to the bath, when tension was stable, PHE was given in a cumulative fashion (10^−9^–10^−5^ M).

The concentration of carotenoids (1.2 × 10^−5^ M) was that described in Mancini *et al.* [23]. Control responses to PHE were compared to the responses obtained in the presence of CCT or crocins, and with those performed after an incubation (20 min) with the NO synthase (NOS) inhibitor, L-NAME (NG-nitro-l-arginine methyl ester, 10^−5^ M), to check the participation of NO, or with the cyclooxygenase (COX) inhibitor, indomethacin (10^−5^ M), to examine the possible involvement of arachidonic acid derivatives.

The data were obtained from the response elicited by aortic rings from at least six rats. For every vascular segment, no more than two consecutive phenylephrine concentration-response curves were performed. For example, a control curve of PHE and a PHE curve in the presence of an inhibitor or carotenoids, separated by a Krebs washing.

### 4.5. Myographical Assays

Assessment of effects of saffron carotenoids on the endothelial modulation of vascular smooth muscle contraction. Intact and de-endothelialized vessels were tested. Vascular segments were de-endothelialized by gently scraping the lumen with a stainless steel wire ground to the inner size of the vessel. PHE (10^−9^–10^−5^ M) was examined in intact and denuded vessels in the absence (control) or presence of crocins or CCT.

Assessment of the role of endothelial prostaglandins on the action of saffron carotenoids. Prostanoid synthesis was inhibited with indomethacin. Cumulative doses of PHE (10^−9^–10^−5^ M) were tested in intact vessels treated with indomethacin (10^−5^ M) in the absence (control) or presence of crocins or CCT.

Assessment of the role of NO on the action of saffron carotenoids. NO synthesis was inhibited with L-NAME. Cumulative doses of PHE (10^−9^–10^−5^ M) were tested in intact vessels treated with L-NAME (10^−5^ M) in the absence (control) or presence of crocins or CCT.

### 4.6. Data Analysis and Statistical Procedures

For every concentration-response curve, two parameters were calculated: the maximum effect (*E*_max_) that the substance being tested elicited and the concentration of this substance which produced 50% of the *E*_max_, (EC_50_), which is expressed as pD_2_ (−log EC_50_). All *E*_max_ and pD_2_ results are shown as the means ± standard error. Statistical significance was determined by Student’s impaired *t* test for two points. All differences were considered significant at a *p* value < 0.05.

### 4.7. Preparation of the Drugs and Chemicals

All compounds different from crocins and CCT samples were from Sigma Aldrich (Alcobendas, Spain). Crocins and CCT were dissolved in the Krebs solution. ACN was obtained from Panreac (Barcelona, Spain), while water was purified through a Milli-Q system (Millipore, Bedford, MA, USA). Indomethacin was prepared in ethanol freshly every experiment. Ethanol was tested in preliminary experiments to rule out toxicity. Other compounds were prepared in distilled water.
